# Endolysins of bacteriophage vB_Sal-S-S10 can naturally lyse *Salmonella enteritidis*

**DOI:** 10.1186/s12917-022-03514-y

**Published:** 2022-11-21

**Authors:** Xinrui Wang, Lili Han, Jiaqing Rong, Huiying Ren, Wenhua Liu, Can Zhang

**Affiliations:** College of Veterinary Medicine, Qingdao Agricultural University, Qingdao, 266109 PR China

**Keywords:** Bacteriophage vB_Sal-S-S10, *Salmonella enteritidis*, Biological and genomic character, Endolysin, Holin, Antibacterial activity

## Abstract

**Background:**

The holin-endolysin lysis system plays an essential role in the phage life cycle. Endolysins are promising alternatives to antibiotics, and have been successfully used against Gram-positive bacteria. However, a few endolysins can externally lyse Gram-negative bacteria, due to the inaccessible peptidoglycan layer covered by the envelope.

**Results:**

This study investigated the lysis system of a new *Siphoviridae* bacteriophage vB_Sal-S-S10 (S10), which, that was isolated from broiler farms, was found to be able to infect 51.4% (37/72) of tested *S. enteritidis* strains. Phage S10 genome had a classic holin-endolysin lysis system, except that one *holin* and one *endolysin* gene were functionally annotated. The *orf 22* adjacent to the lysis cassette was identified as a new *endolysin* gene. Antibacterial activity assays showed that holin had an intracellular penetrating activity against *S. enteritidis* 35; both endolysins acted on the cell envelope of *S. enteritidis* 35 and showed a natural extracellular antibacterial activity, leading to a ~ 1 log titer decrease in 30 min. Protein characterization of lysin1 and lysin2 revealed that the majority of the *N*-terminus and the *C*-terminus were hydrophobic amino acids or positively charged.

**Conclusion:**

In this study, a new *Salmonella* phage vB_Sal-S-S10 (S10) was characterized and showed an ideal development prospect. Phage S10 has a classic holin-endolysin lysis system, carrying an overlapping holin-lysin gene and a novel lysin gene. Both endolysins coded by lysin genes could externally lyse *S. enteritidis*. The natural extracellular antibacterial character of endolysins would provide necessary information for the development of engineering endolysin as the antibiotic alternative against the infection with multidrug-resistant gram-negative bacteria.

**Supplementary Information:**

The online version contains supplementary material available at 10.1186/s12917-022-03514-y.

## Background

*Salmonellosis* is one of the most common foodborne diseases caused by *Salmonella*. With more than 2000 serotypes, *Salmonella* causes a variety of diseases from self-limiting gastroenteritis (nontyphoidal salmonellae) to systemic enteric fever (typhoidal salmonellae), which mainly spread in Africa and South Asia [1]. Globally, more than 93 million cases of salmonellosis are reported each year, causing a significant economic burden [2]. *S. enterica* is the most common *Salmonella* pathogen, infecting many hosts, such as laying hens, pigs, turkeys, and broilers, and easily transferred to humans [3]. Antibiotics have been widely used to prevent and control bacterial diseases since their discovery, but multidrug-resistant bacteria have emerged in recent years, and have rapidly become a global health crisis [4]. *Salmonella* has also shown serious drug resistance, and is on the list of 12 key resistant bacteria published by the WHO (World Health Organization) [5]*.* In particular, China is one of the largest antibiotic consumers, and confronts the challenge of antibiotic resistance [6]. To address the increasing threat of antibiotic resistance, bacteriophages (phages) and their related enzymes have gained increasing attention as antibiotic alternatives [7]. In recent years, phage therapy has been successfully used to control drug-resistant bacterial infections without adverse effects [8].

Phages are viruses that specifically infect bacteria, and are widely distributed in the nature. Over the course of evolution, phages have produced a set of action mechanisms that can lyse host bacteria with high specificity and efficiency [9]. The holin-endolysin system is commonly involved in the phage-mediated lysis of infected bacteria [10]. Phage genomes typically carry at least one set of lysis system. Holin is a small protein (< 150 aa) with at least one transmembrane domain (TMD) and a hydrophilic, highly charged *C*-terminus, which triggers the formation of microholes in the cytoplasmic membrane; active endolysin (lysin) is released from the hole into the periplasm to degrade cell wall peptidoglycan (PG) [11]. Many lysins can degrade the external PGs of gram-positive bacteria and have been widely used as antibacterial agents [12]. However, lysins with natural external lytic activities against Gram-negative bacteria are rarely reported, because the bacterial outer membrane (OM) hinders the permeation of exogenously applied lysins [13]. There have been many attempts to enhance the permeability and activity of lysins, including combining lysins with outer membrane permeabilizers, utilizing protein engineering or encapsulating lysins with other compounds that can penetrate the OM [10, 14]. Several reports have found that some lysins with natural lytic activity against Gram-negative bacteria had a common structural character; they had a cationic or amphipathic region at the C-terminus or N-terminus that facilitated their permeation across the negatively charged OM [14–16].

In this study, a new *Salmonella* phage vB_Sal-S-S10 (S10) was isolated from broiler farms and characterized. Phage S10 has a classic holin-endolysin lysis system with overlapping *holin*-*lysin* genes. Additionally, a novel *lysin* gene was found, and identified to be adjacent to the *holin* gene. Both proteins coded by the predicted *lysin* genes had natural lysis activities against *S. enteritidis*. This study provides new members of the endolysin group that can naturally lyse gram-negative bacteria, and their molecular characteristics provide necessary information for the development of engineering endolysin as antibiotic alternatives in treating multidrug-resistant *S. enteritidis* infection.

## Results

### Morphological characterization of phage S10

**Fig. 1:**
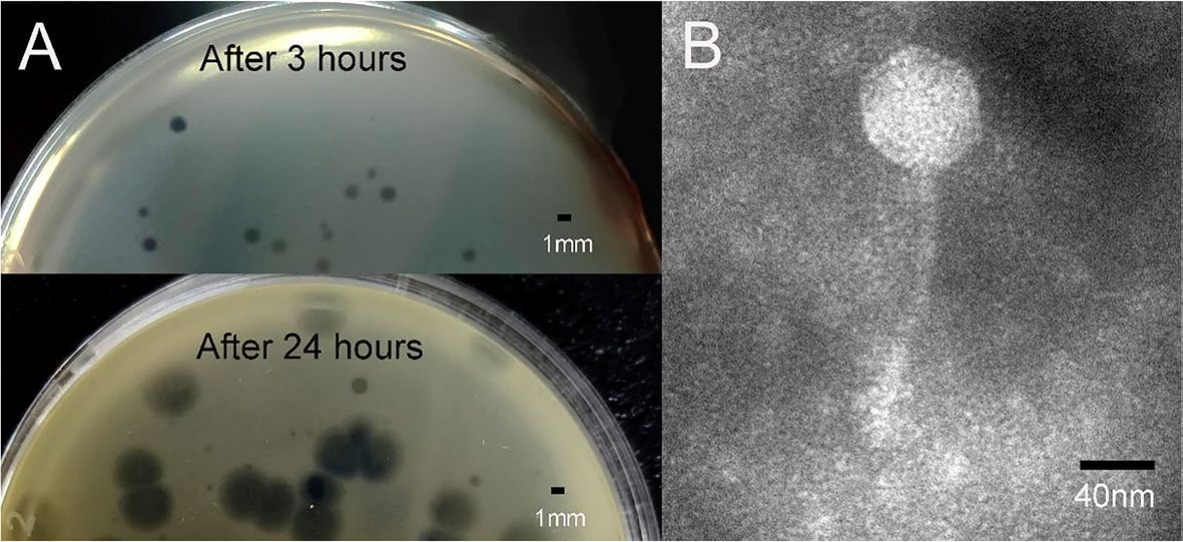
Characterization of phage S10. **A** Phage plaques formed on double-layered agar plates. **B** Morphology of phage S10 in TEM (magnification: × 40.0 K)

Phage S10 formed 1 ~ 2 mm transparent plaques on the lawn of *S. enteritidis* 35 and was surrounded by a growing opaque halo ring that gradually grew to 4 ~ 5 mm (Fig. 1A), suggesting that phage S10 had a depolymerase activity. The transmission electron microscope (TEM) morphology showed that phage S10 had a regular icosahedral head (approximately 72 nm in diameter) and an inextensible tail (approximately 140 nm in length) (Fig. 1B), which indicated that phage S10 belongs to *Caudoviricetes* according to the current guidelines of the International Committee on Taxonomy of Viruses (ICTV).

### Biological characterization of phage S10

Phage S10 had the highest titer of 6.2× 10^10^ pfu/mL on *S. enteritidis* 35 at the optimal MOI of 0.0001 (Table S1). Seventy-two *S. enteritis* strains were used to test the host range of phage S10, 51.3% (37/72) of which could be lysed by phage S10. This result fitted the common view that *Salmonella* phages generally have a broad host range. However, the EOP of phage S10 on the 72 *Salmonella* strains showed obvious phage titer differences from 10^4^ to 10^10^ pfu/ml, which indicated that there was a large different proliferation efficiency of phage S10 on different strains (Table 1). There are complex interactions between phage and host cells, and the defense mechanism of host bacteria may be the reason for such large differences in the proliferation efficiency of phage S10 [17].

**Table 1:** EOP of phage vB_SalS-S10 in 37 *S. enteritidis* strains

Strain numbers	Strain name	Titer phage vB_SalS-S10 (PFU/mL)	The efficiency of plating (EOP)
1	*S. enteritidis* 3	4.86 × 10^9^	2.84
2	*S. enteritidis* 4	2.27 × 10^7^	1.33 × 10^−2^
3	*S. enteritidis* 6	3.84 × 10^7^	2.25 × 10^−2^
4	*S. enteritidis* 7	2.90 × 10^6^	1.70 × 10^− 3^
5	*S. enteritidis* 8	4.76 × 10^7^	2.78 × 10^−2^
6	*S. enteritidis* 9	1.06 × 10^9^	6.20 × 10^−1^
7	*S. enteritidis* 10	2.30 × 10^8^	1.35 × 10^−1^
8	*S. enteritidis* 11	3.10 × 10^8^	1.81 × 10^−1^
9	*S. enteritidis*12	7.20 × 10^7^	4.21 × 10^−2^
10	*S. enteritidis* 13	7.90 × 10^6^	4.62 × 10^−3^
11	*S. enteritidis* 15	1.50 × 10^6^	8.77 × 10^−4^
12	*S. enteritidis* 16	9.00 × 10^4^	5.26 × 10^−5^
13	*S. enteritidis* 17	2.50 × 10^6^	1.46 × 10^−3^
14	*S. enteritidis* 18	8.96 × 10^7^	5.24 × 10^−2^
15	*S. enteritidis* 20	9.40 × 10^9^	5.5
16	*S. enteritidis* 21	3.20 × 10^7^	1.87 × 10^−2^
17	*S. enteritidis* 23	3.30 × 10^6^	1.93 × 10^−3^
18	*S. enteritidis* 24	1.00 × 10^5^	5.85 × 10^−5^
19	*S. enteritidis* 30	8.76 × 10^7^	5.12 × 10^−2^
20	*S. enteritidis* 32	1.01 × 10^7^	5.91 × 10^−3^
21	*S. enteritidis* 34	2.00 × 10^5^	1.17 × 10^−4^
22	*S. enteritidis* 35	1.71 × 10^9^	1
23	*S. enteritidis* 36	5.00 × 10^4^	2.92 × 10^−5^
24	*S. enteritidis* 39	1.90 × 10^8^	1.11 × 10^−1^
25	*S. enteritidis* 41	3.20 × 10^7^	1.87 × 10^−2^
26	*S. enteritidis* 42	2.60 × 10^6^	1.52 × 10^−3^
27	*S. enteritidis* 44	3.00 × 10^4^	1.75 × 10^−5^
28	*S. enteritidis* 45	2.00 × 10^4^	1.17 × 10^−5^
29	*S. enteritidis* 47	8.00 × 10^4^	4.68 × 10^−5^
30	*S. enteritidis* 48	1.80 × 10^6^	1.05 × 10^−3^
31	*S. enteritidis* 49	4.90 × 10^6^	2.87 × 10^−3^
32	*S. enteritidis* 53	1.10 × 10^6^	6.43 × 10^−4^
33	*S. enteritidis* 64	5.00 × 10^4^	2.92 × 10^−5^
34	*S. enteritidis* 65	4.00 × 10^4^	2.34 × 10^−5^
35	*S. enteritidis* 68	4.00 × 10^4^	2.34 × 10^−5^
36	*S. enteritidis* 70	1.10 × 10^6^	6.43 × 10^−4^
37	*S. enteritidis* 71	1.40 × 10^6^	8.19 × 10^−4^

**Fig. 2:**
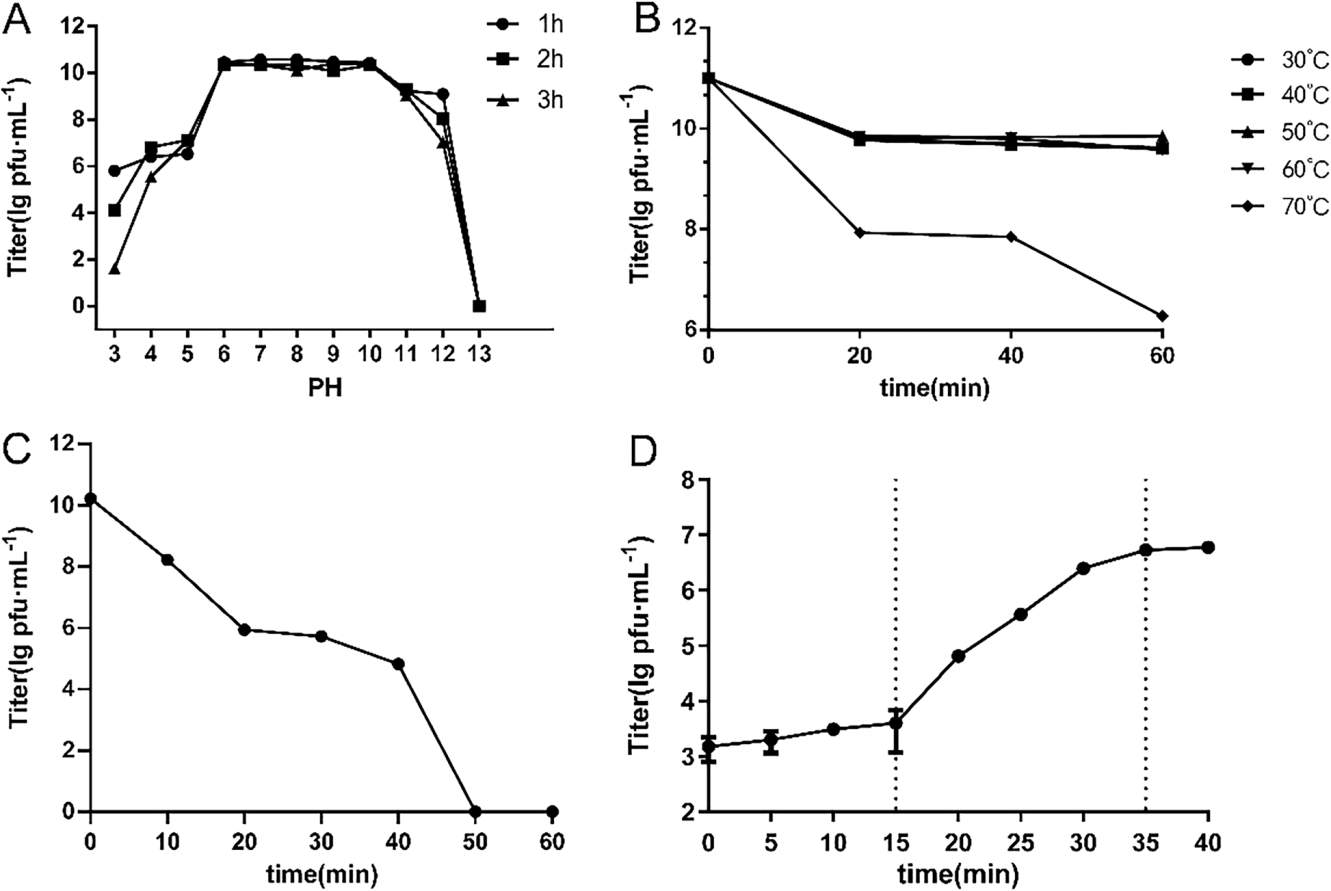
Biological characteristics of phage S10. **A** pH stability. **B** thermal stability. **C** UV stability. **D** One-step growth curve on *S. enterica* 35. The phage S10 data are expressed as the mean ± SD

Phage S10 kept stable in the range of pH value 6 ~ 10 (Fig. 2A) at 30 ~ 60 °C (Fig. 2B). When exposed to UV light, the phage showed continuously decreased titer and was completely inactivated in 50 min (Fig. 2C). The one-step growth assay of phage S10 showed that there was a 15-min latent period, a large number of offspring phages began to release from 35 min, and the burst size was 60 pfu/cell (Fig. 2D).

### Genomic characterization of phage S10

**Fig. 3:**
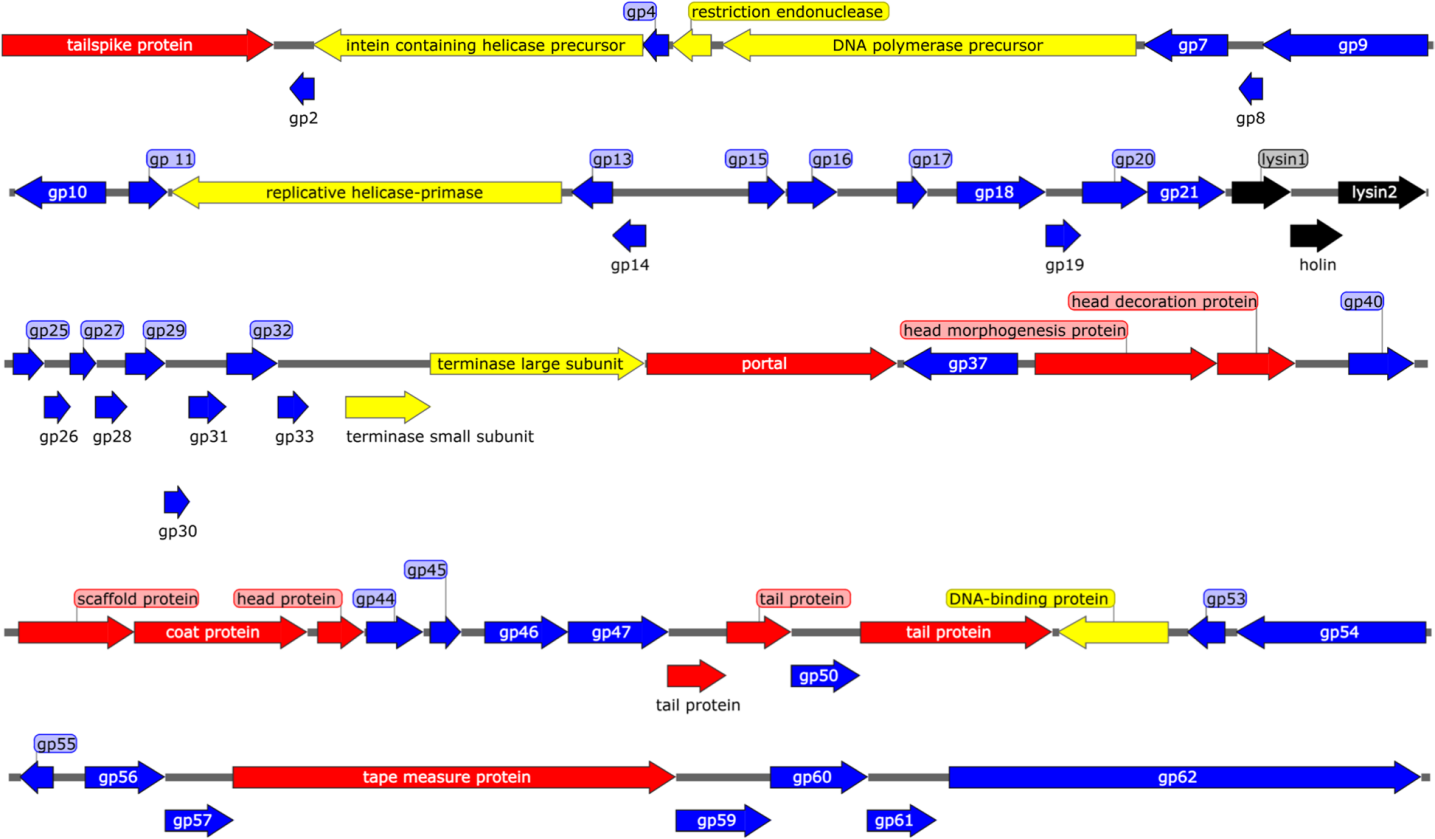
Detailed phage S10 genome annotation. The arrows indicate the direction of transcription of each gene. The colors represent ORFs with different predictive functions; the structural and packaging proteins are marked with red, the lysis-related proteins are marked with black, the proteins associated with transcriptional regulation are marked with yellow, and the hypothetical proteins are marked with blue

The phage S10 genome was sequenced and characterized. The genome was 43,324-bp-long, having a 49.5% GC content. A total of 63 ORFs were predicted, out of which, 46 were positive-stranded, and the rest were negative-stranded. Twenty-two of the 63 ORFs were annotated as functional genes, including 12 structure-related genes, 3 lysis-related genes, and 7 transcription- and replication-related genes (Fig. 3). The tRNAs, virulence genes, or drug-resistance genes were not found in the genome.

Phage S10 had a classic holin-endolysin lysis system. The *orf 23* was annotated as a *holin* gene; three transmembrane domains were predicted using TMHMM-2.0 and were found to belong to type I holin. The *orf 24* was annotated as a *lysin* gene; a CD search and Phyre 2 analysis showed that it belongs to the lyz_endolysin_autolysin family and has glycosidase activity. For *orf 22* prediction, although it had homology with the *holin* gene of the phage Jersey (94.62 identified with 86% coverage), no TMD was found by TMHMM-2.0. This indicated a possible unsure result through homology analysis because there is typically at least one TMD in the holin protein [10]. Also, a CD search and Phyre 2 analysis had not characterized its possible function.

**Fig. 4:**
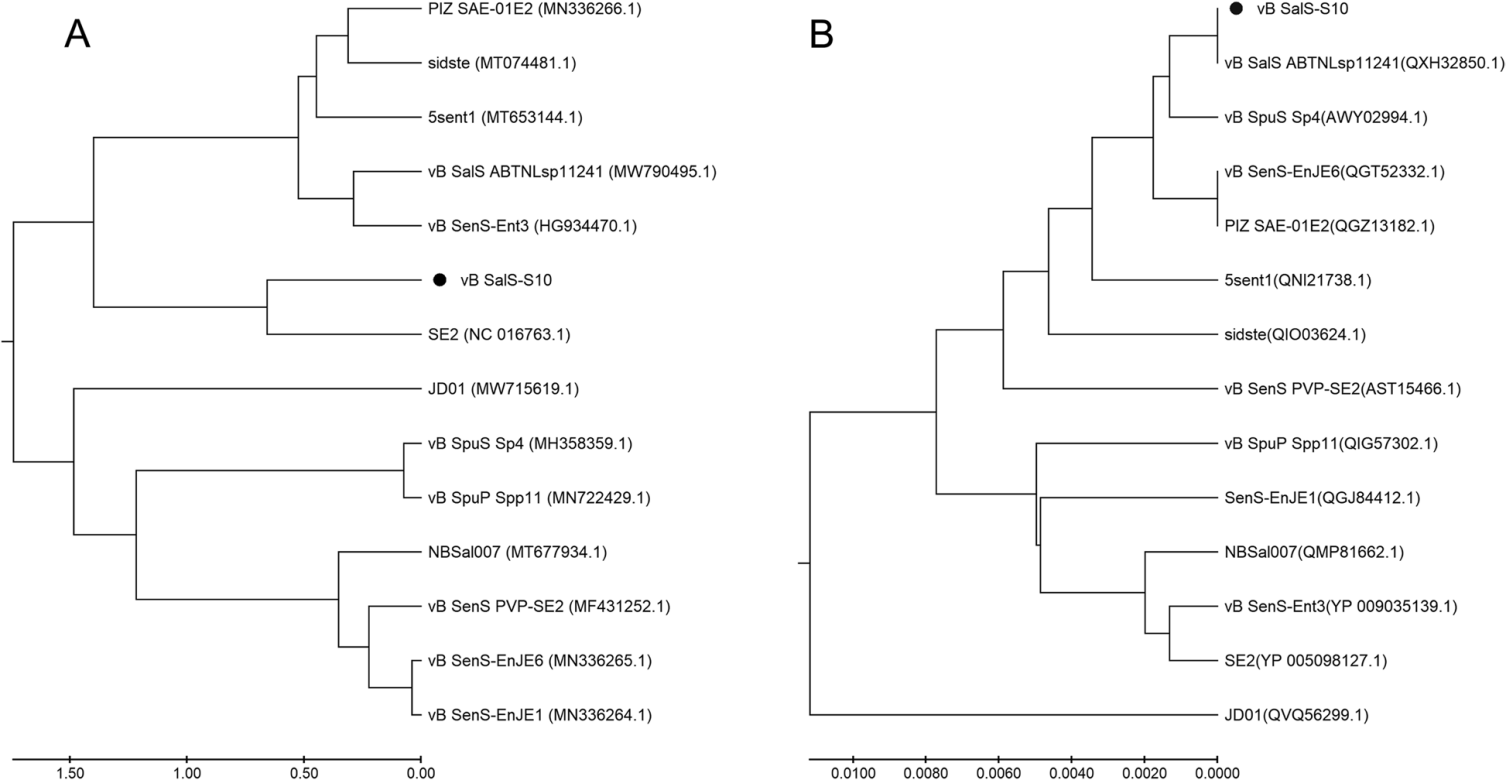
Bioinformatic analyses of the genome of vB_Sal-S-S10. The genomic sequences (**A**) and the terminase large subunit sequences (**B**) were compared in the NCBI GenBank database, and the phylogenetic tree was generated using the neighbor-joining method with default parameters in MEGA 5.0. • Represents phage S10

Phage S10 was homologously analyzed based on the whole genome sequence and terminase large subunit. Thirteen phages with high similarity to phage S10 were selected to construct the evolutionary tree, and all phages belonged to the *Jerseyvirus* genus. At the genomic level, 14 phages were clustered into two groups, and *Salmonella* phage se2 (NC016763.1) had the highest similarity (93.27%) to phage S10, with 89% coverage (Fig. 4A). At the terminase large subunit level, phage S10 and 13 homologous phages showed a highly conserved character (> 99.05%). The evolutionary tree was clustered into two groups, and the *Salmonella* phage ABTNLsp11241 (QXH32850.1) had the highest homology with phage S10 (Fig. 4B).

### Expression and antibacterial activity of lysis-related genes

**Fig. 5:**
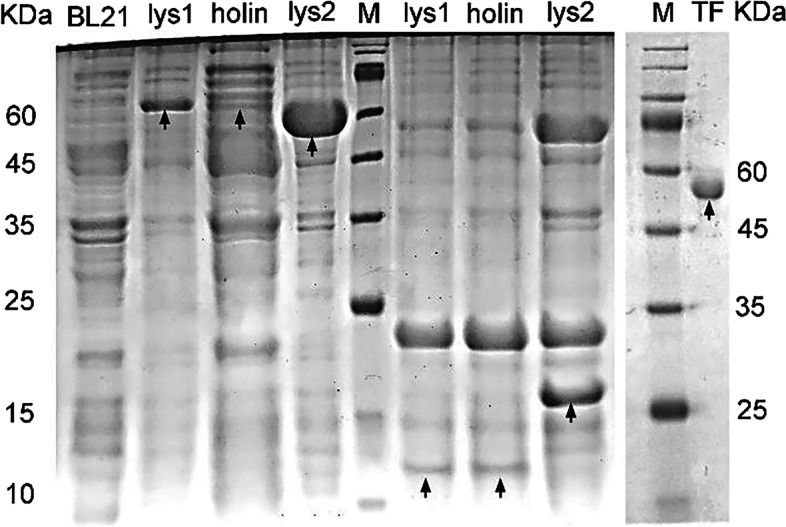
SDS-PAGE analysis of lysin1, holin, and lysin2. *E. coli* BL21 carrying recombinant plasmid (pCold-lysin1, pCold-holin, pCold-lysin2) was induced with 0.5 mM IPTG at 16 °C for 16 h. Ultrasonic purified proteins (left) and purified protein without TF tag (right) in the supernatant were detected by SDS–PAGE (full-length blots are presented in Fig. S5. The samples derive from the same experiment and blots were processed in parallel)

Predicted *lysin1* (*orf 22*), *holin* (*orf 23*), and *lysin2* (*orf 24*) were amplified by PCR to construct recombinant plasmids and expressed in *E. coli* BL21. The SDS–PAGE (Fig. 5) and Western-blot (Fig. S1) results showed that the recombined proteins were separately expressed in soluble forms with mass of 63.96 kD, 62.69 kD and 69.34 kD. The purified proteins were treated with HRV-3C protease, and the 52 KD TF tag and his_6_ tag were removed.

**Fig. 6:**
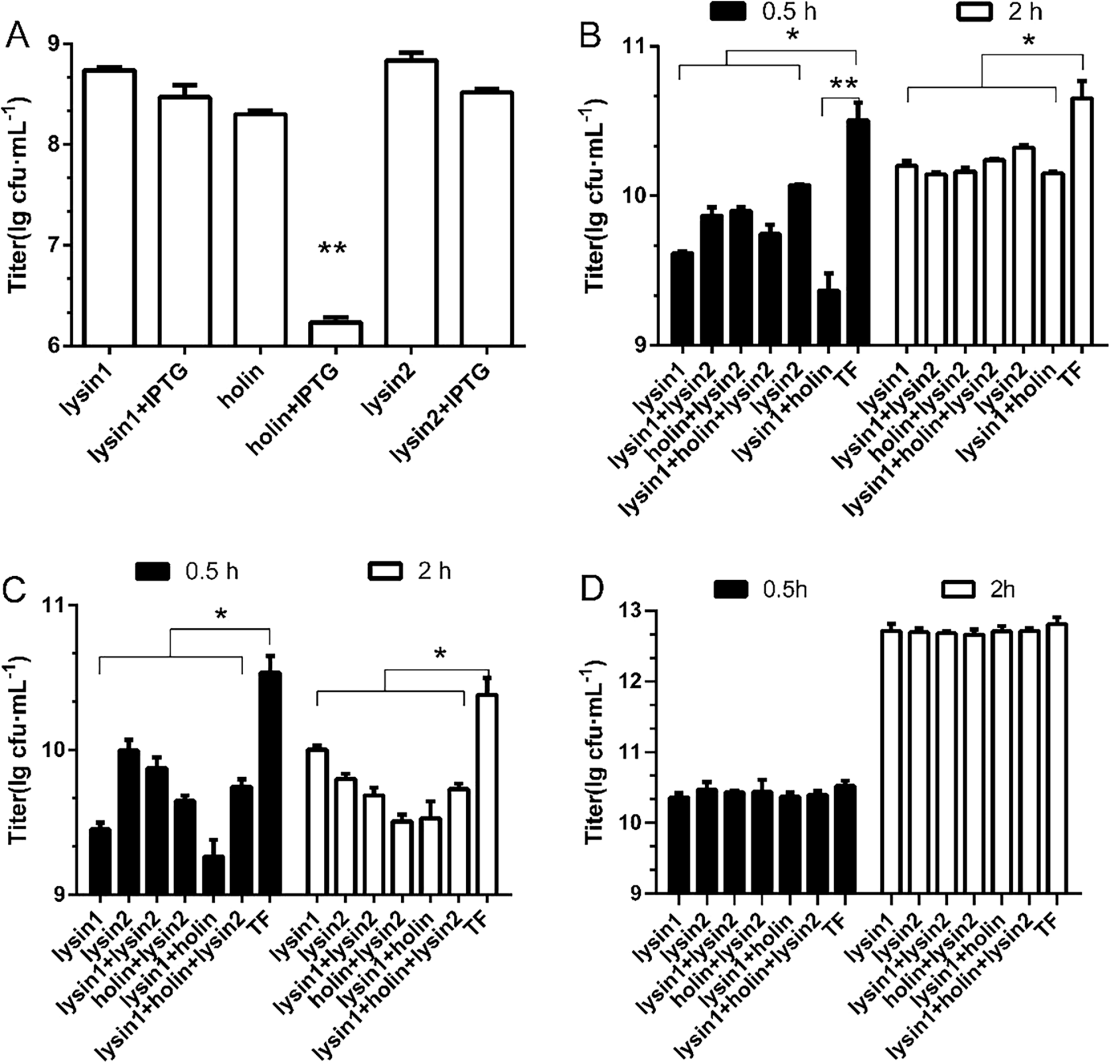
Lytic activity assay of the phage S10 lysis proteins. **A** *E. coli* BL21 carrying recombinant plasmid (pCold-lysin1, pCold-holin, pCold-lysin2) was induced with 0.5 mM IPTG at 16 °C for 16 h. Colony counts were performed at 16 h to test the intracellular lytic activities of lysin1, holin and lysin2*. E. coli* BL21 carrying the pCold-TF plasmid was used as the control. **B** *S. enterica* 35 was treated with lysin1, holin, lysin2 or their mixture, and the titers were assessed at 0.5 h and 2 h to determine the extracellular antibacterial activities of protein. TF protein was used as the control. **C** The effect of lysis proteins against *S. enterica* 35 with EDTA treatment at 0.5 h and 2 h. TF protein was used as the control. **D** The antibacterial activities of lysis proteins against *E. coli* BL21 without EDTA treatment at 0.5 h and 2 h. All assays were repeated three times, and the data are expressed as the mean ± SD

The intracellular antibacterial activity of purified proteins was tested. Compared with the TF control group, the expression of holin (encoded by *orf 23*) led to a 2-log titer reduction at 16 h and showed an obvious perforation effect (*P <* 0.01); lysin1 (encoded by *orf 22*) and lysin2 (encoded by *orf 24*) showed a negative result (*P > 0.05*). This result was consistent with our previous prediction that *orf 22* did not encode a holin protein (Fig. 6A).

**Fig. 7:**
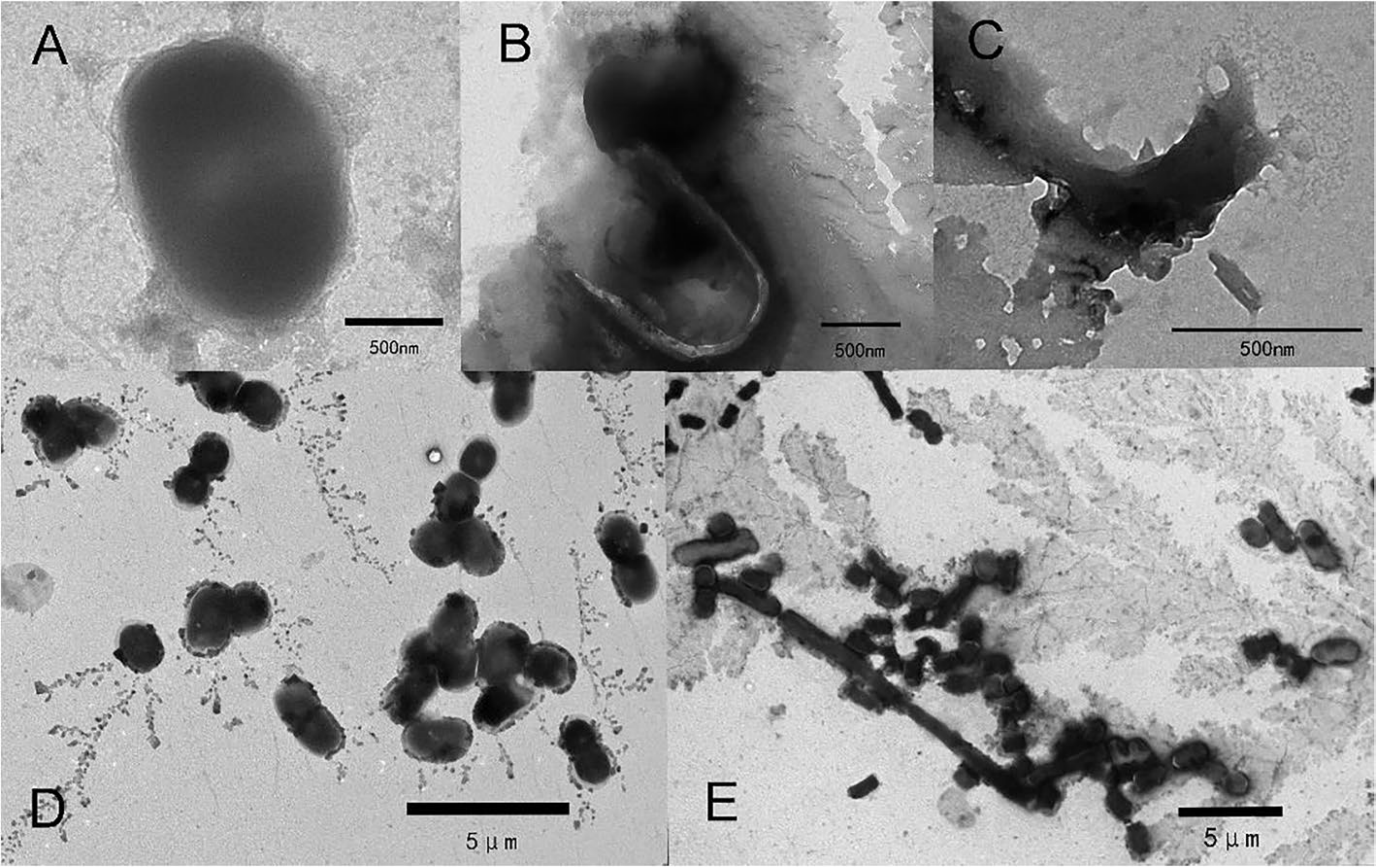
TEM images of *S. enterica* 35 and *E. coli* BL21 treated with lysin proteins. **A** Untreated *S. enterica* 35. **B**-**C** The gradual disintegration of *S. enterica* 35 treated with lysin proteins at 0.5 h. **D** Untreated *E. coli* BL21. **E** Morphological changes from short rods to long rods of *E. coli* BL21 treated with lysin proteins at 0.5 h

To further confirm the role of *orf 22* and *orf 24*, the extracellular antibacterial activities of lysin1 and lysin2 were tested against *S. enterica* 35 and *E. coli* BL21. Both lysin1 and lysin2 showed natural antibacterial activities against *S. enterica* 35. The bacterial titer had decreased by approximately 1 log at 30 min and still had a 4-fold decrease at 2 h (Fig. 6B). Ethylene diamine tetraacetic acid (EDTA) reportedly can assist endolysin in passing through the Gram-negative bacterial outer membrane to allow efficient peptidoglycan hydrolysis [18]. The coeffect of lysins with EDTA showed a consistent result, compared with the groups without EDTA treatment, all of the groups with EDTA showed better antibacterial activities against *S. enterica* 35 (*P <* 0.05) (Fig. 6C). Morphological observation by TEM revealed a process of the gradual disintegration of *S. enterica* 35 (Fig. 7A-C).

To determine the specificity of lysin1 and lysin2, their antibacterial activities were also evaluated in *E. coli* BL21. However, the results were different from the *S. enterica* 35 findings. Compared with the TF control group, lysin1 and lysin2 showed no antibacterial activity (Fig. 6D), indicating the species specificity of these phage endolysins. Interestingly, the morphologies of single colonies treated with either lysin1 or lysin2 were significantly smaller than that of the control group (Fig. S2). The morphological changes of the bacteria were further observed with TEM. Treating *E. coli* BL21 with lysins caused a morphological change from short rods to long rods (Fig. 7D-E). This curious phenomenon may suggest that lysins inhibit the division of *E. coli* BL21.

The co-effects of lysin1, holin and lysin2 against *S. enterica* 35 were also evaluated. The results showed that the holin-lysin mixture had a better antibacterial effect than that of the single protein treatment (*P < 0.05*). Lysin1 + holin had the strongest antibacterial effect (Fig. 6B, C). The holin and lysin proteins of phage S10 had synergistic effects on host cells, consistent with previous reports [19]. Also, the released ATP level of *S. enterica* 35 with or without lysin treatment were tested to reveal the changes of the membrane permeability. Compared with the control group, there was a significantly ATP increasing in lysin-treated groups, which indicated that lysin1 and lysin2 had the extracellular activities against *S. enterica* 35 (Fig. S3).

## Discussion

Bacteriophages have developed a variety of lysis strategies during the evolutionary process, and holin-endolysin is the most common lysis system [10]. This study identified and systematically analyzed the holin-endolysin lysis system of phage S10, which could infect and inactivate *S. enterica*.

Currently known holin-lysis systems are encoded by *holin*, *endolysin* and *Rz/Rz1* (or *spanin*) genes. They can independently act on the inner membrane, peptidoglycan layer and outer membrane of host cell. *Holin* and *lysin* are indispensable and directly involved in the induction of cell lysis [10]. Commonly, the lysis cassettes are clustered in the genomes of most dsDNA phages and are frequently contiguous. In fact, when an ORF adjacent to an identifiable *endolysin* gene, codes for a small (< 150 aa) putative product with the potential to form at least one TMD, it is frequently assigned as the *holin* gene [20]. Phages usually encode endolysin or holin as a single gene, and some phages had more than one gene with a complex organization strategy [21]. For the lytic cassette of phage S10, three accessory lysis proteins encoded by *orf 22*, *orf 23*, and *orf 24* were predicted by a BLAST sequence analysis in the NCBI GenBank database.

There was partial overlap between *orf 23* and *orf 24*, encoding the holin and lysin proteins, respectively; this is a common phenomenon in the phage genome, where holin and lysin often exist as fully or partially overlapping genes [22]. Holin (*orf 23*) was predicted to have three TMDs and is a type I holin, as previously described [23]. The bacteriostasis experiments confirmed its intracellular degradation function. Over 50 unrelated gene families encode holins, making them the most diverse group of proteins with common functions [24]. No conserved domain was found in the holin protein of phage S10 by CD search, indicating that it is a newly identified member of the holin family.

Interestingly, most homologous sequences with *orf 22* in the database had no functional annotation except the holin of the phage jersey (YP008239771.1). It is known that holins have at least one TMD and a highly charged, hydrophilic, *C-*terminal domain. The *C-*terminus sequence analysis of *orf 22* revealed a similar character, which suggested that *orf 22* encoded a holin protein, but a further TMD analysis showed that there was no TMD in the protein encoded by *orf 22*; thus, additional experimental analysis is needed to confirm the role of *orf 22*. Three accessory lysis proteins of phage S10 were expressed in a soluble form in *E. coli* BL21 to conduct the bacteriostasis test. The activities of the protein coded by *orf 22* against *S. enterica 35* were tested with or without EDTA. EDTA is commonly used as permeabilizer on Gram-negative bacteria, which can combine with divalent cations to increase the fluidity and permeability of cell membrane. The results showed that the protein coded by *orf 22* had no intracellular activity, but it did have obvious extracellular antibacterial activity with or without EDTA, which indicates that it plays an endolysin role; thus, it was annotated as the *lysin1* gene although no functional domain was predicted. Interestingly, lysin1 and lysin2 all showed natural antibacterial activity against *S. enterica* 35, and as predicted, there was a co-effects with EDTA. Recently, limited lysins have been reported to have bactericidal activity against Gram-negative bacteria [14], and most of them have a positively charged hydrophobic amino acid at the *N*- or *C*-terminus [12]. In this study, sequence analysis of lysin1 and lysin2 showed that the majority of the *N*-terminus and the *C*-terminus were hydrophobic amino acids or positively charged (Fig. S4). Endolysins are categorized into different types based on their mode of action and individual enzymatic specificities [25]. Lysin2 of phage S10 has glycosidase activity, but the mechanism of lysin1 activity remains unknown. The genome contained two lysins with different enzymatic activities, which may explain the high titer and efficient lysis ability of phage S10.

Endolysins typically have a broader host range than that of phage against Gram-negative bacteria; they can even be active against multiple genera externally with the help of penetrating agents [26]. In this study, lysin1 and lysin2 showed no lysis ability towards *E. coli* BL21. However, single colonies treated with lysins had a smaller size, which implied that phage S10 lysins affected the growth of *E. coli* BL21. TEM observations showed that treatment of *E. coli* BL21 with lysins led to a morphological change from short rods to long rods. It has been reported that the expression of phage endolysins with TMDs plays a key role in cell elongation, which might enable a large release of phage from the bacteria [27, 28]. Therefore, we propose that the lysins of phage S10 play a similar role in *E. coli* BL21 and inhibited cell division.

The holin and two lysins of phage S10 were shown to be necessary for the completion of host cell lysis, and there is likely an interaction between these proteins. This assumption was verified by the coeffect of the phage S10 lysis proteins on host cells. The mixture of holin and lysins had better lytic activity against *S. enterica* 35 than that of the single proteins; however, the mixture of lysin1 and lysin2 showed no synergistic effect, regardless of whether holin was added. We propose that the phage S10 lysins pass through the outer membrane to hydrolyze the peptidoglycans of the host bacteria, which allows holin easily to pass through the envelope and to form holes in the inner membrane, resulting in highly efficient host cell lysis.

## Conclusion

In conclusion, the new virulent *Siphoviridae* phage S10 produced a high titer and showed good tolerance to physical and chemical factors. It carries two endolysins with natural extracellular antibacterial activities against *S. enteritidis*, and one of them is identified as a new member of the endolysin group. These results would provide necessary information to develop engineering endolysin as the antibiotic alternative against Gram-negative bacterial infection.

## Methods

### Samples and Bacteria strains in this study

Fecal samples were collected from broiler farms in Shandong, China. A total of 72 *S. enteritidis* clinical isolates were randomly selected for the host range test (Table S2). Bacteria strains were cultured in Luria-Bertani (LB) medium at 37 °C overnight and stored at 4 °C until use. All strains were isolated from broiler chickens from different farms at different times, and then identified by 16S rRNA gene sequencing performed by Tsingke Biotechnology Co., Ltd. (Beijing, China). The sensitivity of 72 *S. enteritidis* strains to 8 antibiotics were tested using the Kirby-Bauer method recommended by National Committee for Clinical Laboratory Standards (NCCLS).

### Phage isolation

The *S. enteritidis* 35 strain was used as the host cell for phage isolation. Phages in fecal samples were enriched and isolated by traditional methods as previously described [29]. Briefly, fecal samples were mixed in 500 ml of freshly prepared *S. enteritidis* 35 proliferation solution and incubated at 37 °C overnight. The mixture was then centrifuged at 12,000×g for 30 min, and the supernatant was filtered through a 0.22 μm filter. The presence of phage in the filtrated samples was verified by the traditional double-layer agar method. The plates were cultured at 37 °C for 6 h. A single plaque was selected and purified four times. The isolated phage was designated phage S10 and stored at − 80 °C in 30% glycerol until use.

### Morphology observation of phage S10

The morphology of phage S10 was observed by transmission electron microscopy (HT7700, Hitachi, Japan). Briefly, 10 μL of phage suspension (10^10^ pfu/mL) was transferred onto a carbon-coated grid and stained with 2% uranyl acetate for 5 min. The dimensions of individual phages were measured, and the virion particle size was determined from at least 10 measurements.

### Host range and EOP analysis

The host range of phage S10 was tested with 72 isolated *S. enteritidis* strains using the double-layer agar method. Additionally, the efficiency of plating (EOP) was determined based on the host range test results. The EOP of phage S10 on the *S. enteritidis* 35 strain was set as 1, and the *ratio* of the phage titer on other host strains to that on *S. enteritidis* 35 strain was defined as the EOP of phage S10.

### Stability assay of phage S10

The tolerance of phage S10 to temperature, pH and UV was tested by the double-layer agar method. Phage S10 (10^10^ pfu/ml) was cultured at different temperatures (30, 40, 50, 60 and 70 °C), different pH values (3 to 13) and UV light (the phage was 50 cm away from the UV light). Samples were collected every 20 min under the different temperature conditions, every 1 h under the different pH conditions, and every 10 min under UV. The pH- and UV-treated phage experiments were performed at room temperature. The titers were tested to evaluate phage tolerance. Each experiment was performed three times.

### MOI assay of phage S10

The MOI assay was performed to determine the highest phage proliferation. Phage S10 and host *S. enteritidis* 35 were diluted in multiple ratios and cocultured in different MOIs (0.0001, 0.001, 0.01, 0.1, 1 and 10) at 37 °C for 4 h. *S. enteritidis* 35 cultured without phage S10 was used as the control. The S10 phage titers were tested by the double-layer agar method, and the optimal MOI was determined. The experiments were performed in triplicate.

### One-step growth of phage S10

To further understand the reproductive characteristics of phage S10, the latent period and burst size were determined as previously described [30]. In brief, phage S10 suspensions (10^6^ pfu/mL) were mixed with host *S. enteritidis* 35 (10^7^ cfu/mL) and incubated at 37 °C for 5 min. The mixture was centrifuged at 12,000×g for 1 min to remove unabsorbed phages in the supernatant. The precipitate was resuspended in 7 mL of LB and incubated at 37 °C for 40 min. Two hundred microliter samples were taken every 5 min. The phage titer was determined by the double-layer agar method. Each assay was repeated three times.

### Sequencing and genomic analysis of phage S10

Phage DNA extraction was performed as previously described [30]. The purified genomic DNA of phage S10 was sequenced on an Illumina HiSeq platform (Shenzhen Huitong Biotechnology Co., Ltd., China), and contigs were assembled using the de novo assembly algorithm Newbler version 3.0 with default parameters. GeneMarks (http://topaz.gatech.edu/GeneMark/) and RAST (https://rast.nmpdr.org/) were used for putative open reading frame (ORF) prediction and annotation. Potential tRNA detection was performed using tRNAscan-SE (http://lowelab.ucsc.edu/tRNAscan-SE/). The potential resistance genes and virulence genes were searched in the Antibiotic Resistance Genes Database (http://www.cbcb.umd.edu/publications/ardb-antibiotic-resistance-genes-database) and Comprehensive Antibiotic Resistance Database (https://card.mcmaster.ca/). The predicted holin and lysin proteins of phage S10 were analyzed by CD Search (https://www.ncbi.nlm.nih.gov/Structure/cdd/wrpsb.cgi) to find conserved regions; the transmembrane region was analyzed by TMHMM-2.0 (https://services.healthtech.dtu.dk/service.php?TMHMM-2.0), and the structural character was predicted by Phyre 2 (http://www.sbg.bio.ic.ac.uk/phyre2). For phylogenetic analysis, the phage S10 genome sequence was submitted to NCBI GenBank and searched in the BLAST database (https://blast.ncbi.nlm.nih.gov/Blast.cgi). Thirteen homologous phages were selected to construct a phylogenetic tree based on the genomic analysis and the terminase large subunit using the neighbor-joining method with default parameters in MEGA 5.0. The genome sequence of phage S10 was deposited in GenBank under accession number OL770276.

### Antibacterial activity determination of lysis system

The predicted genes *orf 22* (lysin1), *orf 23* (holin), and *orf 24* (lysin2) were amplified from extracted phage S10 genomic DNA by PCR using designed primers (Table S3) to construct the recombinant plasmids pColdTF-lysin1, pColdTF-holin, and pColdTF-lysin2 using ClonExpress II One-step cloning Kit (Vazyme, Nanjing). The plasmids were transformed into *E coli* BL21. *E. coli* BL21 carrying recombinant plasmid was cultured in 100 ml of LB at 37 °C to the logarithmic growth phase, induced by the addition of 0.5 mM IPTG, and further incubated at 16 °C for 16 h to express the proteins. The fusion proteins were purified and identified as previously reported [31]. In brief, the culture was centrifuged at 4000×g for 10 min, the precipitation was washed three times and resuspended with PBS (pH 7.2) and treated ultrasonically for 30 min (3 s pulse, 1 s pause). Samples were centrifuged with 10,000×g for 10 min, and the presence of the fusion proteins in the supernatant was confirmed by 12% SDS-PAGE. The soluble expressed proteins were purified using a His-tagged Protein Purification Kit (CoWin Biosciences, Beijing) as the manufacturer description and detected by Western-blot, the anti-His tag monoclonal antibody (Solarbio, Beijing) was used as the primary Ab, and the HRP-labeled goat-anti-mouse IgG (Solarbio, Beijing) as the secondary Ab. The identified proteins were treated with HRV 3C protease (sangon biotech, Shanghai) to remove the trigger factor (TF) tag of the pCold-TF vector and recovered using a His-tagged Protein Purification Kit (CoWin Biosciences, Beijing).

Both intracellular and extracellular antibacterial activity assays of the proteins were performed. The intracellular antibacterial activity of holin and lysins against *E. coli* BL21 was tested by the colony counting method after 0.5 mM IPTG induction for 16 h. *E. coli* BL21 carrying the pCold-TF plasmid was used as a control. The extracellular antibacterial activities of the lysins against *S. enteritidis* 35 and *E. coli* BL21 were determined. Briefly, the host bacteria *S. enteritidis* 35 was cultured in LB medium at 37 °C to logarithmic growth phase and centrifuged with 4000×g for 10 min. The precipitate was washed three times and diluted to 2 × 10^3^ cfu/ml with Tris-HCl buffer (pH 7.2). 100 μl *S. enteritidis* 35 (10^3^ cfu/ml) and 100 μl lysin proteins (2 μM) were mixed and cultivated at 37 °C for 2 h. The bacterial titers at 0.5 h and 2 h were determined by the colony count method, and TF protein was used as the control. Also, the morphological changes of the bacterial samples treated with lysin1 and lysin2 were observed by TEM. Briefly, bacterial cells were collected by centrifugation at 5000×g for 5 min, and the bacterial precipitates were washed three times and resuspended in 20 μL Tris-HCl buffer (pH 7.2). The samples were transferred to carbon-coated grid, followed by TEM observation. The coeffects of lysin proteins with EDTA (2 μM) were also evaluated against *S. enteritidis* 35 (10^3^ cfu/ml) as described above, and TF protein was used as a control. In addition, the expressed proteins (2 μM) were mixed (lysin1 + holin, lysin1 + lysin2, holin+lysin2, lysin1 + holin+lysin2), and the extracellular antibacterial activity was assessed as described above to verify the synergistic effects on host cells. All of the experiments were repeated three times.

The membrane permeability of *S. enteritidis* 35 treated by lysin1 or lysin2 were tested by the extracellular ATP tests. The *S. enteritidis* 35 (10^7^ cfu/ml) solution was mixed with lysin1 (2 μM) or lysin2 (2 μM), and incubated at 37 °C for 30 min. The TF protein was used as the control. The ATP content was determined by ATP detection kit (Solarbio, Beijing). All experiments were repeated three times.

### Statistical analysis

The data was analyzed using the GraphPad Prism Version 6.02 software and shown as mean ± SD. The difference between two groups was compared by Student’s *t*-test and the differences among more than two groups were compared by one-way ANOVA. A value of *p* < 0.05 was considered to be statistically significant. The complete data are included in Data S1.

## Supplementary Information

**Additional file 1.**

**Additional file 2.**

**Additional file 3.**

**Additional file 4.**

**Additional file 5.**

**Additional file 6.**

**Additional file 7.**

**Additional file 8.**

**Additional file 9.**

**Additional file 10.**

## Data Availability

All data generated or analysed during the study are included within the article and supplemental material.
