# Supplementary material for: Endolysins of bacteriophage vB_Sal-S-S10 can naturally lyse Salmonella enteritidis
Source: BMC Vet Res. 2022 Nov 21;18:410. doi: 10.1186/s12917-022-03514-y (PMC9677904; doi:10.1186/s12917-022-03514-y)
Supplement: Supplementary file 1 — Additional file 1. [file 12917_2022_3514_MOESM1_ESM.docx]

**Table S1**

**The best multiplicity of infection(MOI)of phage vB_SalS-S10**

| S. enteritidis 35(cfu/ml) | Titer phage vB_SalS-S10 (pfu/mL) | MOI | Three hours later(pfu/mL) |
| --- | --- | --- | --- |
| 1×10^9^ | 1×10^10^ | 10 | 3.2×10^9^ |
| 1×10^9^ | 1×10^9^ | 1 | 3.2×10^9^ |
| 1×10^9^ | 1×10^8^ | 0.1 | 1.38×10^10^ |
| 1×10^9^ | 1×10^7^ | 0.01 | 4.6×10^10^ |
| 1×10^9^ | 1×10^6^ | 0.001 | 5.67×10^10^ |
| 1×10^9^ | 1×10^5^ | 0.0001 | 6.2×10^10^ |
