# Supplementary material for: Endolysins of bacteriophage vB_Sal-S-S10 can naturally lyse Salmonella enteritidis
Source: BMC Vet Res. 2022 Nov 21;18:410. doi: 10.1186/s12917-022-03514-y (PMC9677904; doi:10.1186/s12917-022-03514-y)
Supplement: Supplementary file 2 — Additional file 2. [file 12917_2022_3514_MOESM2_ESM.docx]

**Table S2**

**Drug susceptibility of 72 *S. enteritidis* strains**

| Strain name | Susceptibility results | | | | | | | | ST type | Separation time | Region of isolation |
| --- | --- | --- | --- | --- | --- | --- | --- | --- | --- | --- | --- |
|  | AMP | DO | FFC | ENR | CIP | CIN | NEO | C |  |  |  |
| *S. enteritidis* 1 | R | R | R | I | S | S | R | I | 11 | 2013 | Weifang |
| *S. enteritidis* 2 | R | R | R | R | S | I | R | I | 11 | 2013 | Weifang |
| *S. enteritidis* 3 | R | R | R | R | S | S | R | I | 11 | 2013 | Yantai |
| *S. enteritidis* 4 | R | R | R | S | I | R | R | S | 11 | 2013 | Yantai |
| *S. enteritidis* 5 | R | R | S | R | I | S | R | S | 11 | 2013 | Linyi |
| *S. enteritidis* 6 | R | R | S | R | I | I | R | S | 11 | 2013 | Linyi |
| *S. enteritidis* 7 | R | R | S | R | R | I | R | I | 11 | 2014 | Rizhao |
| *S. enteritidis* 8 | R | R | I | R | R | I | R | S | 11 | 2014 | Linyi |
| *S. enteritidis* 9 | R | R | S | R | R | I | I | S | 11 | 2015 | Weifang |
| *S. enteritidis* 10 | R | R | R | S | R | I | I | S | 11 | 2015 | Weifang |
| *S. enteritidis* 11 | R | R | R | R | R | S | I | R | 11 | 2015 | Yantai |
| *S. enteritidis* 12 | R | R | R | I | R | S | S | R | 11 | 2016 | Yantai |
| *S. enteritidis* 13 | R | I | R | R | S | I | S | R | 11 | 2016 | Weihai |
| *S. enteritidis* 14 | R | I | R | R | R | I | S | S | 11 | 2016 | Weihai |
| *S. enteritidis* 15 | R | I | S | R | R | I | S | S | 11 | 2016 | Jining |
| *S. enteritidis* 16 | R | R | R | R | R | I | R | R | 11 | 2016 | Jining |
| *S. enteritidis* 17 | R | R | R | I | R | S | R | I | 11 | 2017 | Yantai |
| *S. enteritidis* 18 | R | R | R | R | R | S | R | S | 11 | 2017 | Linyi |
| *S. enteritidis* 19 | R | R | R | I | S | S | R | S | 11 | 2017 | Weihai |
| *S. enteritidis* 20 | R | R | I | S | S | I | R | S | 11 | 2017 | Jining |
| *S. enteritidis* 21 | R | R | I | R | I | I | R | R | 11 | 2017 | Rizhao |
| *S. enteritidis* 22 | R | R | R | R | I | I | R | R | 11 | 2017 | Linyi |
| *S. enteritidis* 23 | R | I | R | R | S | I | I | R | 11 | 2017 | Weifang |
| *S. enteritidis* 24 | R | R | R | I | R | I | I | R | 11 | 2017 | Yantai |
| *S. enteritidis* 25 | R | R | R | I | S | R | I | S | 2441 | 2017 | Yantai |
| *S. enteritidis* 26 | R | R | R | S | R | R | I | S | 2441 | 2017 | Linyi |
| *S. enteritidis* 27 | R | R | R | S | R | I | I | S | 11 | 2017 | Linyi |
| *S. enteritidis* 28 | R | R | R | R | I | I | I | S | 305 | 2017 | Rizhao |
| *S. enteritidis* 29 | R | R | R | R | I | I | I | S | 367 | 2017 | Rizhao |
| *S. enteritidis* 30 | R | R | S | R | I | I | S | R | 11 | 2017 | Rizhao |
| *S. enteritidis* 31 | R | R | R | R | S | I | S | R | 11 | 2017 | Yantai |
| *S. enteritidis* 32 | R | R | R | R | S | S | S | R | 2441 | 2017 | Linyi |
| *S. enteritidis* 33 | R | R | R | R | R | S | I | R | 2441 | 2017 | Linyi |
| *S. enteritidis* 34 | R | R | R | I | R | S | I | S | 11 | 2017 | Weifang |
| *S. enteritidis* 35 | R | R | R | R | R | S | I | S | 11 | 2017 | Yantai |
| *S. enteritidis* 36 | R | R | R | S | R | I | I | S | 6022 | 2017 | Yantai |
| *S. enteritidis* 37 | R | R | R | S | R | I | I | R | 13 | 2017 | Weifang |
| *S. enteritidis* 38 | R | R | I | I | S | I | I | R | 11 | 2017 | Yantai |
| *S. enteritidis* 39 | R | I | R | R | S | I | R | R | 11 | 2017 | Yantai |
| *S. enteritidis* 40 | R | I | R | R | S | I | R | S | 11 | 2017 | Weihai |
| *S. enteritidis* 41 | R | R | R | R | R | I | R | S | 11 | 2019 | Weihai |
| *S. enteritidis* 42 | R | R | R | R | R | I | R | S | 11 | 2019 | Jining |
| *S. enteritidis* 43 | R | R | R | I | R | I | R | I | 321 | 2019 | Jining |
| *S. enteritidis* 44 | R | R | R | S | I | I | R | I | 11 | 2019 | Weifang |
| *S. enteritidis* 45 | R | R | S | R | I | I | I | S | 5106 | 2019 | Yantai |
| *S. enteritidis* 46 | R | R | R | R | I | I | I | S | 11 | 2019 | Yantai |
| *S. enteritidis* 47 | R | I | R | R | S | S | I | S | 11 | 2019 | Linyi |
| *S. enteritidis* 48 | R | R | R | R | S | S | I | S | 11 | 2019 | Linyi |
| *S. enteritidis* 49 | R | R | R | R | S | S | I | S | - | 2019 | Rizhao |
| *S. enteritidis* 50 | R | R | R | I | R | S | I | R | 11 | 2019 | Rizhao |
| *S. enteritidis* 51 | R | R | R | S | R | S | I | R | 11 | 2019 | Rizhao |
| *S. enteritidis* 52 | R | R | R | S | R | R | I | R | 11 | 2019 | Linyi |
| *S. enteritidis* 53 | R | R | I | S | R | R | S | I | 11 | 2019 | Weifang |
| *S. enteritidis* 54 | R | R | R | I | R | I | S | S | 11 | 2019 | Yantai |
| *S. enteritidis* 55 | R | R | R | I | R | I | S | S | 11 | 2019 | Yantai |
| *S. enteritidis* 56 | R | R | R | I | R | I | S | R | 11 | 2019 | Weifang |
| *S. enteritidis* 57 | R | R | R | R | R | I | S | R | 11 | 2019 | Weifang |
| *S. enteritidis* 58 | R | R | R | R | S | S | R | S | 11 | 2019 | Weifang |
| *S. enteritidis* 59 | R | R | R | R | S | I | R | S | 5166 | 2019 | Jining |
| *S. enteritidis* 60 | R | R | R | R | I | I | R | S | - | 2019 | Jining |
| *S. enteritidis* 61 | R | R | R | R | I | S | R | S | 11 | 2019 | Weifang |
| *S. enteritidis* 62 | R | I | R | R | I | I | R | I | 40 | 2019 | Yantai |
| *S. enteritidis* 63 | R | I | R | R | S | I | I | S | 17 | 2019 | Rizhao |
| *S. enteritidis* 64 | R | R | R | R | S | I | I | I | 92 | 2021 | Linyi |
| *S. enteritidis* 65 | R | R | R | R | R | I | I | R | 2441 | 2021 | Weifang |
| *S. enteritidis* 66 | R | R | S | I | R | I | I | R | 11 | 2021 | Weifang |
| *S. enteritidis* 67 | R | R | R | S | R | S | I | S | - | 2021 | Yantai |
| *S. enteritidis* 68 | R | R | R | I | R | S | I | S | 40 | 2021 | Rizhao |
| *S. enteritidis* 69 | R | R | R | R | R | R | S | R | 11 | 2021 | Linyi |
| *S. enteritidis* 70 | R | R | R | R | R | S | S | R | 34 | 2021 | Weifang |
| *S. enteritidis* 71 | R | I | R | S | R | I | I | I | 34 | 2021 | Weifang |
| *S. enteritidis* 72 | R | R | R | R | R | I | I | I | 11 | 2021 | Linyi |

Note: AMP, Ampicillin; DO, Doxycycline; FFC, Florfenicol; ENR, Enrofloxacin; CIP, Ciprofloxacin; CIN, Cinoxacin; NEO, Neomycin; C, Chloramphenicol; R, resistance; I, intermediate; S, sensitive; -, No corresponding MLST typing was found.
