# Supplementary material for: Endolysins of bacteriophage vB_Sal-S-S10 can naturally lyse Salmonella enteritidis
Source: BMC Vet Res. 2022 Nov 21;18:410. doi: 10.1186/s12917-022-03514-y (PMC9677904; doi:10.1186/s12917-022-03514-y)
Supplement: Supplementary file 3 — Additional file 3. [file 12917_2022_3514_MOESM3_ESM.docx]

**Table S3**

**primers used for phage vB_SalS-S10 gene cloning**

| Name | Primer sequences (5’-3’) | Reaction conditions  (Tm) | Amplified fragment length (bp) |
| --- | --- | --- | --- |
| *Lysin1* | F:ggtatcgaaggtaggcatatgATGTTAATCTCCGAAGGTA  R:agcagagattacctatctagaTTATTTCCCCCACGGTAACTT | 64℃ | 327 |
| *holin* | F:ggtatcgaaggtaggcatatgATGCTCAACTCACAAATCGG  R:agcagagattacctatctagaTCACTGATGTTTCGGTTTGA | 63℃ | 291 |
| *lysin2* | F:ggtatcgaaggtaggcatatgATGTCAAACCGAAACATCAGT  R:agcagagattacctatctagaCTACTTCGCAGCGCGCCCT | 65℃ | 489 |

Note: F, forward primer; R, Lower case represents the homologous sequence at the end of the vector, upper case represents the specific amplified sequence.
