# Supplementary material for: Endolysins of bacteriophage vB_Sal-S-S10 can naturally lyse Salmonella enteritidis
Source: BMC Vet Res. 2022 Nov 21;18:410. doi: 10.1186/s12917-022-03514-y (PMC9677904; doi:10.1186/s12917-022-03514-y)
Supplement: Supplementary file 4 — Additional file 4. [file 12917_2022_3514_MOESM4_ESM.docx]

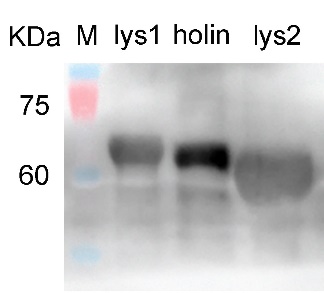


**Figure S1. Western-blot results of lysin1, holin and lysin2(full-length blots are presented in Figure S6).**
