# Supplementary material for: Endolysins of bacteriophage vB_Sal-S-S10 can naturally lyse Salmonella enteritidis
Source: BMC Vet Res. 2022 Nov 21;18:410. doi: 10.1186/s12917-022-03514-y (PMC9677904; doi:10.1186/s12917-022-03514-y)
Supplement: Supplementary file 5 — Additional file 5. [file 12917_2022_3514_MOESM5_ESM.docx]

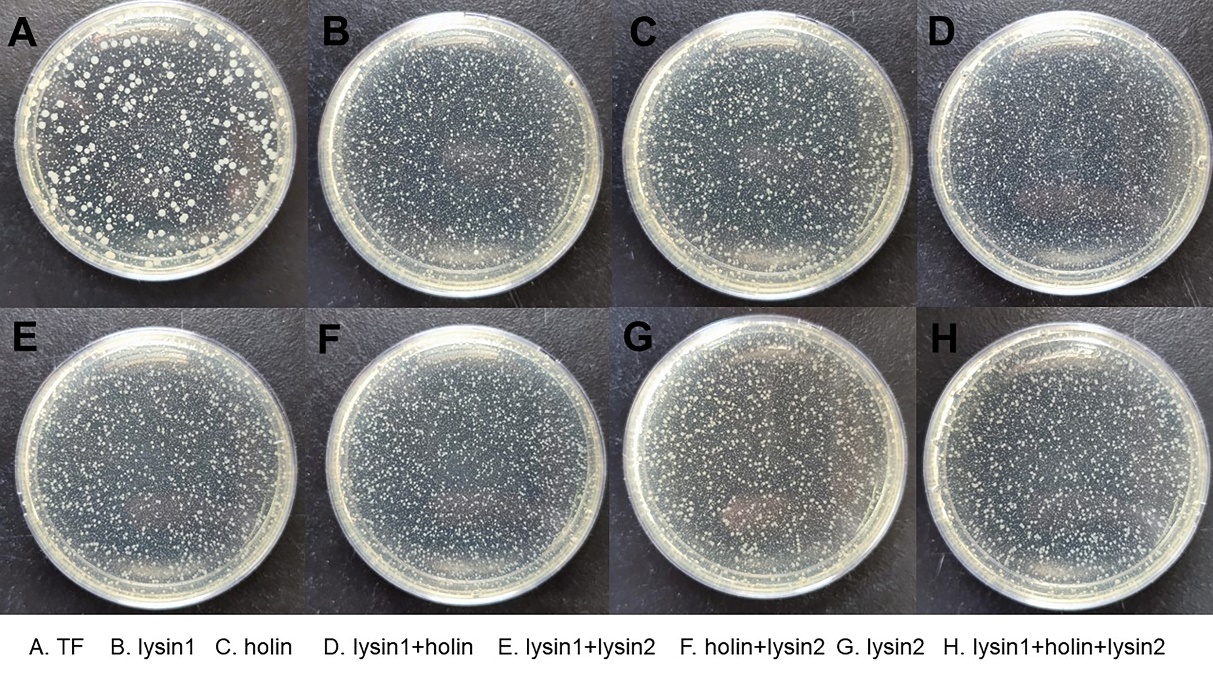


**Figure S2. Treatment of *E. coli* BL21 with lysin1, holin and lysin2.**

(A) TF treatment. (B) Lysin1 treatment. (C) holin treatment. (D) Lysin1+holin treatment. (E) Lysin1+Lysin2 treatment. (F) holin+Lysin2 treatment. (G) Lysin2 treatment. (H) Lysin1+holin+Lysin2 treatment.
