# Supplementary figures and images for: Endolysins of bacteriophage vB_Sal-S-S10 can naturally lyse Salmonella enteritidis
Source: BMC Vet Res. 2022 Nov 21;18:410. doi: 10.1186/s12917-022-03514-y (PMC9677904; doi:10.1186/s12917-022-03514-y)

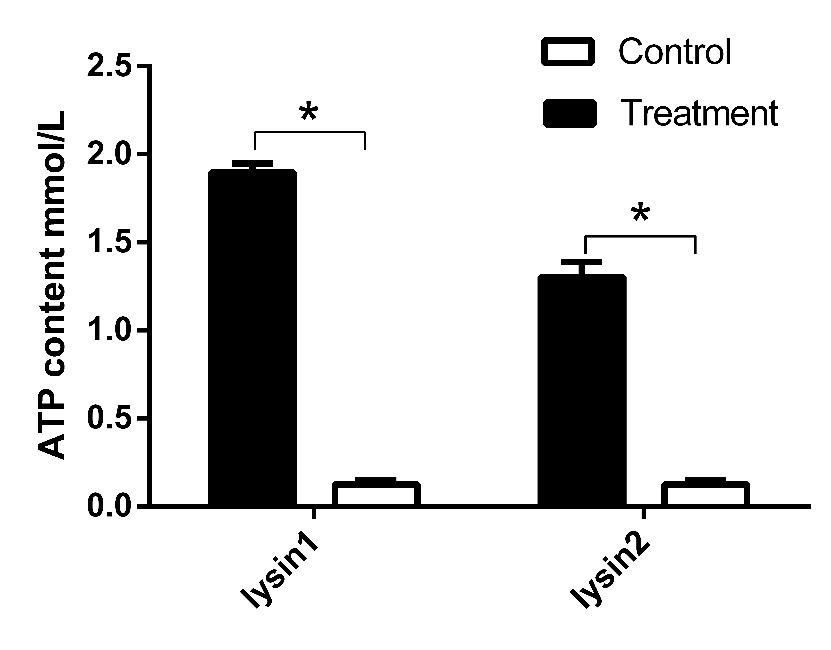


**Figure S3. Effects of lysin1 and lysin2 on ATP content in bacterial fluids**

Supplement: Supplementary file 6 — Additional file 6. [file 12917_2022_3514_MOESM6_ESM.docx]

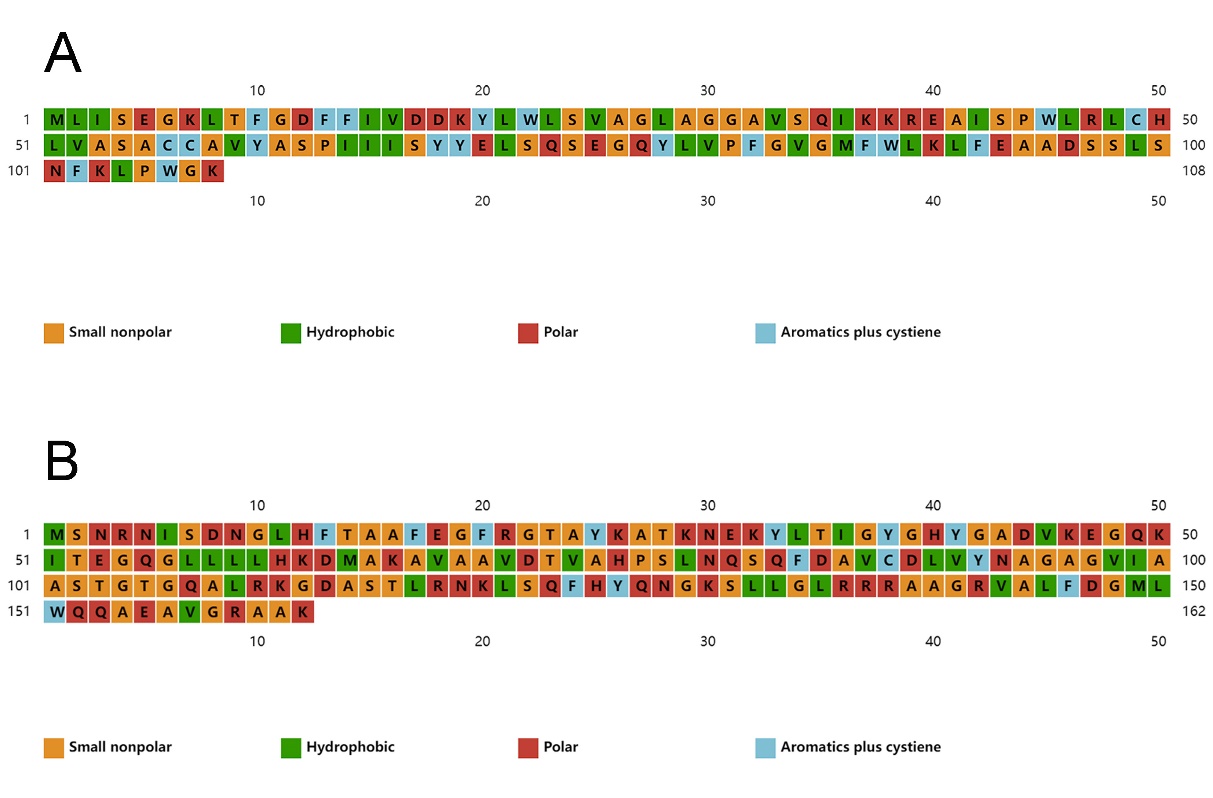


**Figure S4.** **Protein sequence analysis.**

(A) lysin1 sequence analysis. (B) lysin2 sequence analysis.

Supplement: Supplementary file 7 — Additional file 7. [file 12917_2022_3514_MOESM7_ESM.docx]

**Figure 5 unprocessed versions**


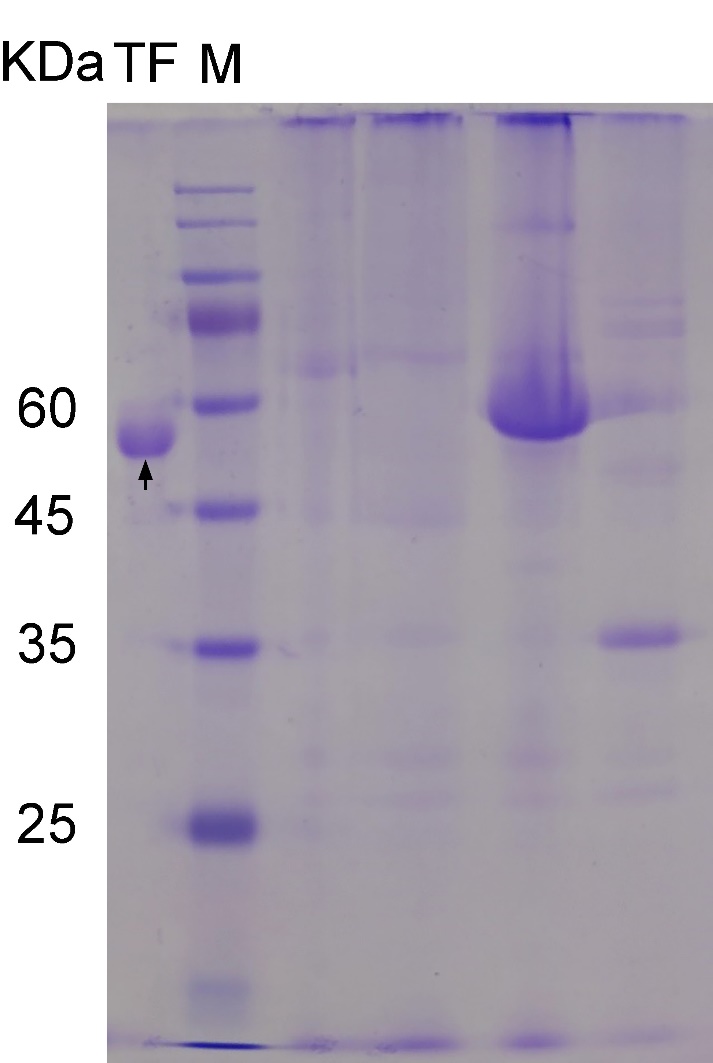

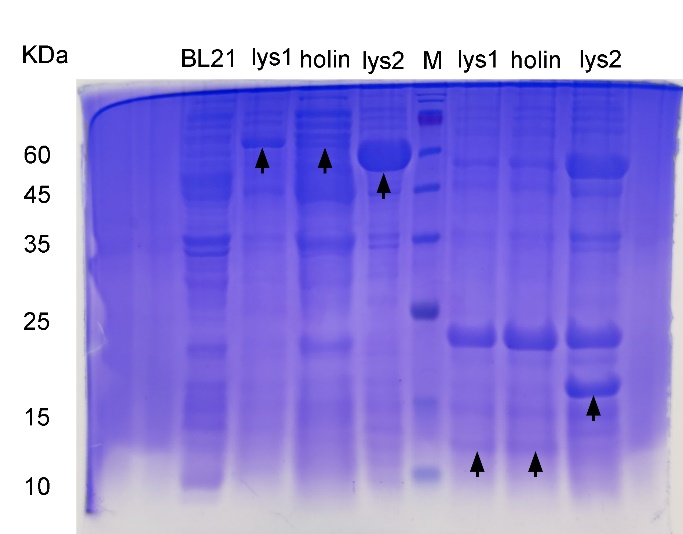

Supplement: Supplementary file 8 — Additional file 8. [file 12917_2022_3514_MOESM8_ESM.docx]

**Figure S1 unprocessed versions**


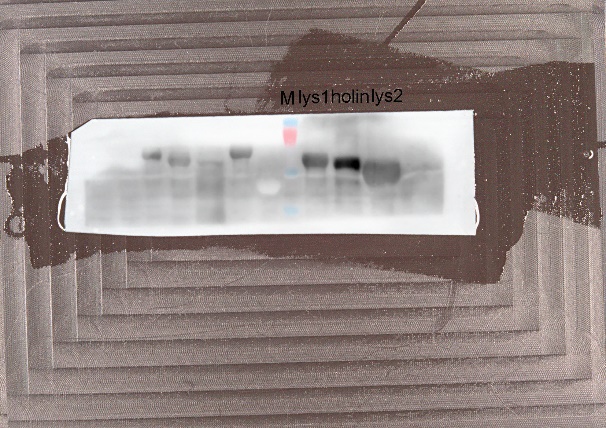

Supplement: Supplementary file 9 — Additional file 9. [file 12917_2022_3514_MOESM9_ESM.docx]
